# *Fusobacterium nucleatum* Secretes Outer Membrane Vesicles and Promotes Intestinal Inflammation

**DOI:** 10.1128/mBio.02706-20

**Published:** 2021-03-02

**Authors:** Melinda A. Engevik, Heather A. Danhof, Wenly Ruan, Amy C. Engevik, Alexandra L. Chang-Graham, Kristen A. Engevik, Zhongcheng Shi, Yanling Zhao, Colleen K. Brand, Evan S. Krystofiak, Susan Venable, Xinli Liu, Kendal D. Hirschi, Joseph M. Hyser, Jennifer K. Spinler, Robert A. Britton, James Versalovic

**Affiliations:** a Department of Pathology and Immunology, Baylor College of Medicine, Houston, Texas, USA; b Department of Pathology, Texas Children’s Hospital, Houston, Texas, USA; c Department of Molecular Virology and Microbiology, Baylor College of Medicine, Houston, Texas, USA; d Department of Pediatrics, Baylor College of Medicine, Houston, Texas, USA; e Department of Surgical Sciences, Vanderbilt University Medical Center, Nashville Tennessee, USA; f Department of Pediatrics, Texas Children’s Cancer Center, Texas Children’s Hospital, Houston, Texas, USA; g Department of Cell and Developmental Biology, Vanderbilt University, Nashville, Tennessee, USA; h Department of Pharmacological and Pharmaceutical Sciences, University of Houston, Houston, Texas, USA; i Department of Pediatrics and Human and Molecular Genetics, Children’s Nutrition Research Center, Baylor College of Medicine, Houston, Texas, USA

**Keywords:** *Fusobacterium nucleatum*, enteroids, organoids, inflammation, outer membrane vesicles, TLR4, epithelium, intestine, microbiome

## Abstract

Multiple studies have implicated microbes in the development of inflammation, but the mechanisms remain unknown. Bacteria in the genus *Fusobacterium* have been identified in the intestinal mucosa of patients with digestive diseases; thus, we hypothesized that Fusobacterium nucleatum promotes intestinal inflammation. The addition of >50 kDa F. nucleatum conditioned media, which contain outer membrane vesicles (OMVs), to colonic epithelial cells stimulated secretion of the proinflammatory cytokines interleukin-8 (IL-8) and tumor necrosis factor (TNF). In addition, purified F. nucleatum OMVs, but not compounds <50 kDa, stimulated IL-8 and TNF production; which was decreased by pharmacological inhibition of Toll-like receptor 4 (TLR4). These effects were linked to downstream effectors p-ERK, p-CREB, and NF-κB. F. nucleatum >50-kDa compounds also stimulated TNF secretion, p-ERK, p-CREB, and NF-κB activation in human colonoid monolayers. In mice harboring a human microbiota, pretreatment with antibiotics and a single oral gavage of F. nucleatum resulted in inflammation. Compared to mice receiving vehicle control, mice treated with F. nucleatum showed disruption of the colonic architecture, with increased immune cell infiltration and depleted mucus layers. Analysis of mucosal gene expression revealed increased levels of proinflammatory cytokines (KC, TNF, IL-6, IFN-γ, and MCP-1) at day 3 and day 5 in F. nucleatum-treated mice compared to controls. These proinflammatory effects were absent in mice who received F. nucleatum without pretreatment with antibiotics, suggesting that an intact microbiome is protective against F. nucleatum-mediated immune responses. These data provide evidence that F. nucleatum promotes proinflammatory signaling cascade*s* in the context of a depleted intestinal microbiome.

## Abstract (precis)

Several studies have identified an increased abundance of *Fusobacterium* in the intestinal tracts of patients with colon cancer, liver cirrhosis, primary sclerosing cholangitis, gastroesophageal reflux disease, HIV infection, and alcoholism. However, the direct mechanism(s) of action of *Fusobacterium* on pathophysiological within the gastrointestinal tract is unclear.

## INTRODUCTION

Recently, it has been hypothesized that the oral cavity may serve as a reservoir for potential pathobionts that can exacerbate intestinal disease (1–4). In support of this hypothesis, increased abundances of oral microbes, including *Fusobacterium* spp, have been reported in the intestines of patients with colon cancer, primary sclerosing cholangitis, gastroesophageal reflux disease, HIV infection, alcoholism, and inflammatory bowel disease (IBD) (2, 5–21). In patients with IBD, an increased abundance of *Fusobacterium* spp. has been identified in biopsy specimens (6, 10–20), and the presence of *Fusobacterium* strongly correlates with disease status (6). *Fusobacterium* is an anaerobic, Gram-negative opportunistic pathogen from the *Fusobacteriaceae* family that can cause several human diseases, including periodontal disease, intrauterine infection, Lemierre’s syndrome, skin ulcers, and appendicitis (22–28). Of the *Fusobacterium* species, F. nucleatum has recently emerged as a compelling candidate for causing human diseases given its prevalence in tissue specimens (10, 11, 14). In colorectal cancer, F. nucleatum promotes a NF-κB-driven proinflammatory genetic signature, including tumor necrosis factor (TNF) and interleukin-6 (IL-6) gene expression (29, 30), cytokines that are also important in intestinal inflammation. Despite the relative abundance of *Fusobacterium* species in gastrointestinal diseases, the literature to date has focused on intestinal F. nucleatum in colorectal cancer. Whether F. nucleatum is also a driver of intestinal inflammation in the normal gut represents a major gap in knowledge.

Liu et al. demonstrated that F. nucleatum produces outer membrane vesicles (OMVs) (31), nanoparticles that are naturally secreted by Gram-negative bacteria. OMVs typically contain antigenic components that can activate Toll-like receptors (TLRs) on epithelial cells or immune cells. TLR activation is linked to activation of the NF-κB pathway and elicitation of proinflammatory cytokine release. In the APC^Min/+^ colorectal cancer model, F. nucleatum potentiates intestinal tumorigenesis via a TLR4 signaling cascade (32). However, the link between F. nucleatum, OMVs, TLR4, and NF-κB activation in the noncancerous intestinal epithelium has not yet been fully addressed. Here, we connected these concepts and demonstrated that F. nucleatum produced OMVs activated TLR4 to drive extracellular signal-regulated kinase (ERK), CREB, NF-κB, and proinflammatory cytokines in human cell lines and human colonoid monolayers. We also identified a role for F. nucleatum in initiating colonic inflammation in mice harboring a human microbiome.

## RESULTS

### *F. nucleatum* subspecies *polymorphum* adheres to intestinal mucus and secretes OMVs.

**FIG 1:**
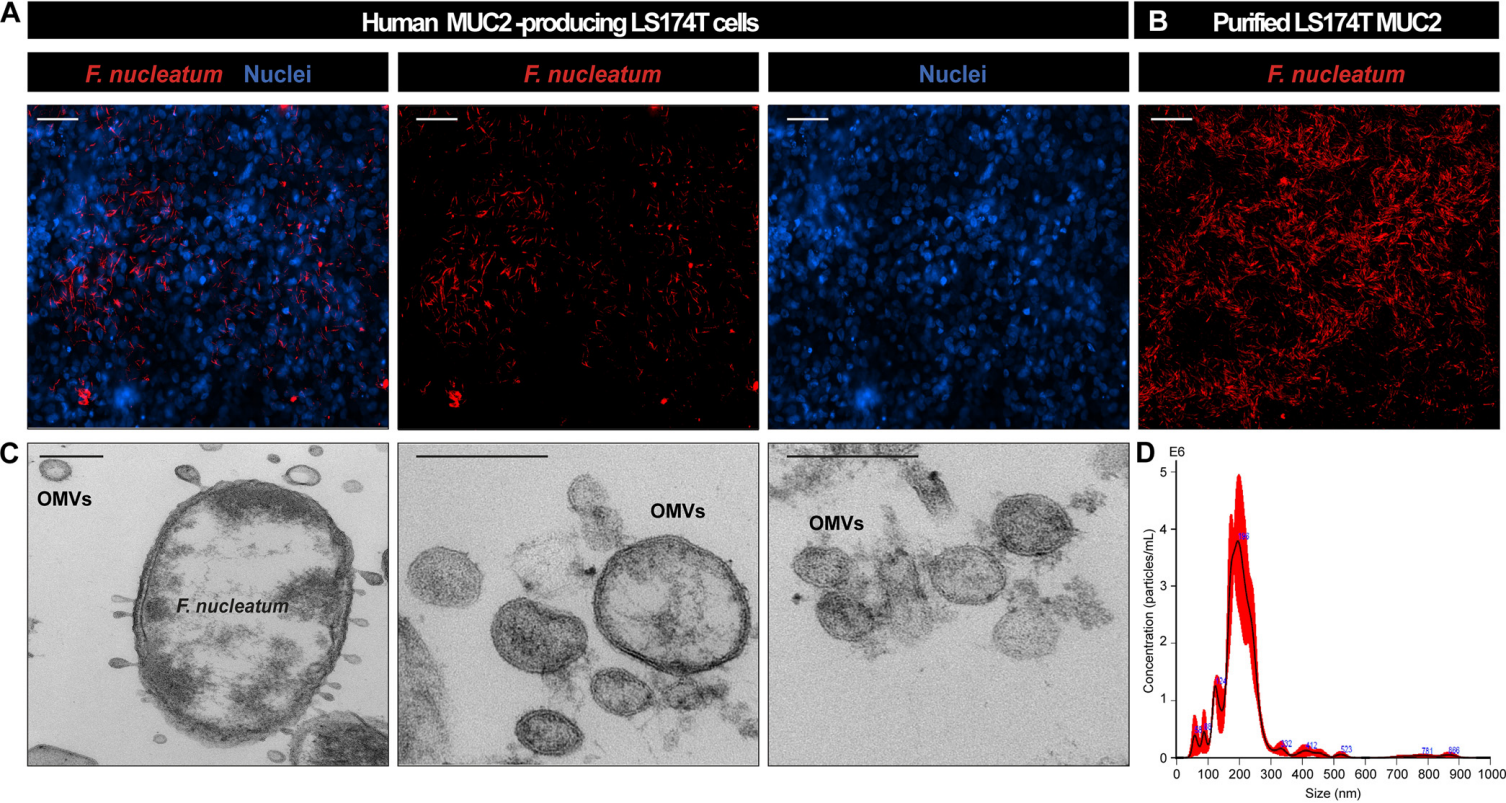
F. nucleatum subsp. *polymorphum* adheres to colonic MUC2 and secretes OMVs. (A) Representative images of T84 cells after incubation with fluorescently tagged F. nucleatum subsp. *polymorphum* counterstained with nuclear dye Hoechst (scale bar, 50 μm). (B) Representative image of fluorescently tagged F. nucleatum subsp. *polymorphum* adhered to purified MUC2 (scale bar, 50 μm). (C) TEM images of F. nucleatum (cross-section) with OMVs attached and surrounding the bacterium. Images on the right-hand side depict the various sizes of OMVs (scale bar, 200 nm). (D) Nanoparticle tracking analysis of F. nucleatum subsp. *polymorphum* OMVs.

Several studies have identified increased abundances of the oral microbe *Fusobacterium* in setting of colorectal cancer (29, 33–35), liver cirrhosis (36–38), primary sclerosing cholangitis (39–41), gastroesophageal reflux disease (42–46), HIV infection (47–49), alcoholism (50), and IBD (6, 10–19). Given the prevalence of F. nucleatum in mucosal specimens, we tested the hypothesis that this pathobiont could promote an epithelial proinflammatory response and potentially contribute to intestinal inflammation. Using fluorescently tagged F. nucleatum subsp. *polymorphum* ATCC 10953, we found that F. nucleatum resided in aggregates in the mucus layer adjacent to human colonic T84 cells (Fig. 1A). To confirm binding to the mucus layer, we also examined adhesion of F. nucleatum to coverslips coated with purified MUC2 from T84 cells and observed robust adhesion (Fig. 1B). Colonization of the intestinal mucus layer allows microbes such as F. nucleatum to secrete host-modulating subcellular structures or compounds in close proximity to the epithelium. One potential subcellular structure that could influence the host is the OMV. Previous groups have shown that F. nucleatum subsp. *nucleatum* and F. nucleatum subsp. *animalis* can secrete OMVs (31, 51). Consistent with these findings, we observed F. nucleatum subsp. *polymorphum* secreted a range of OMVs, with an average hydrodynamic diameter of 212 ± 7 nm, as determined by NanoSight (Fig. 1C and D).

### *F. nucleatum* subsp. *polymorphum* secreted compounds and purified OMVs promote secretion of colonic proinflammatory cytokines.

**FIG 2:**
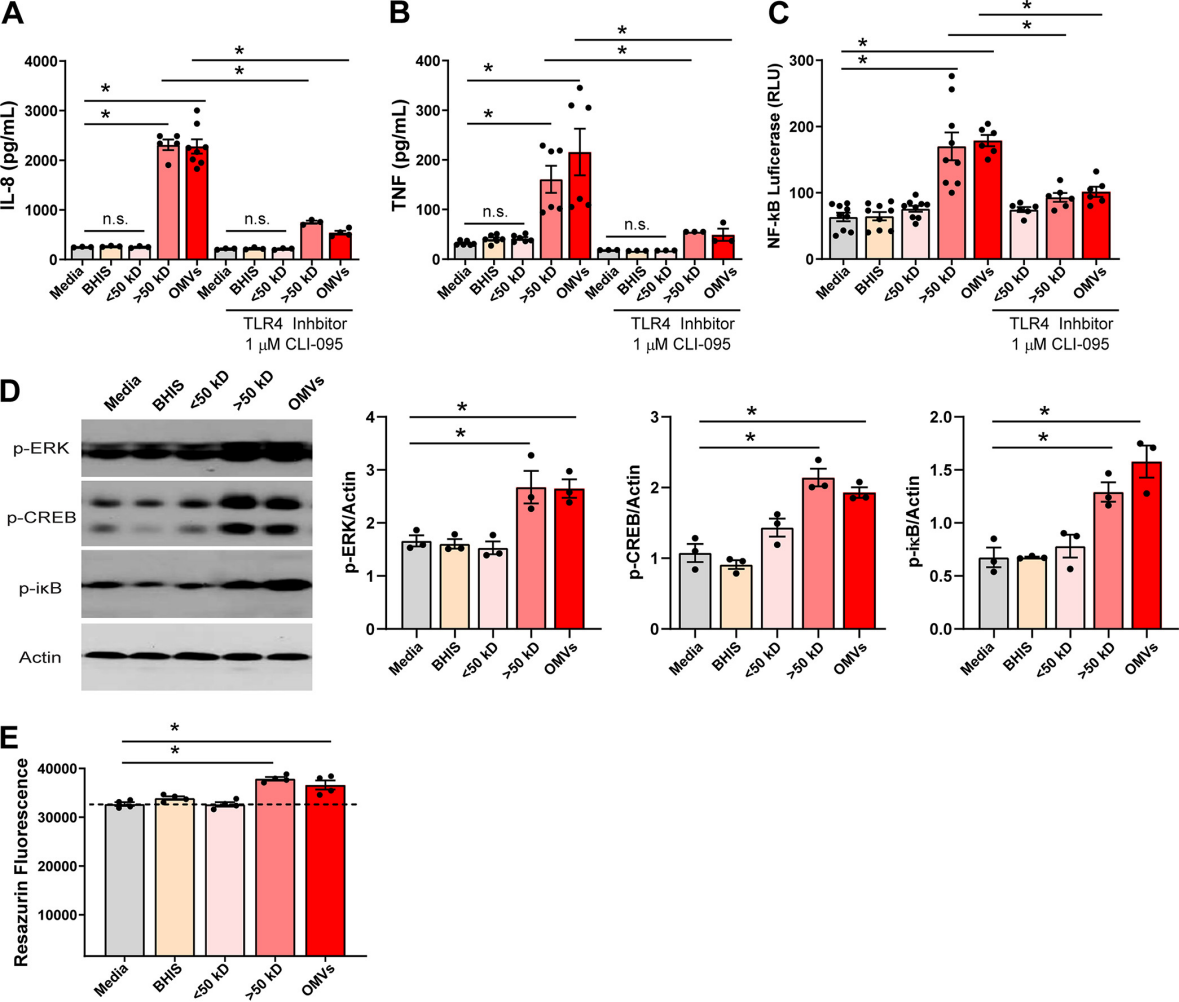
F. nucleatum compounds and OMVs promote IL-8, TNF, NF-κB, and MAPK activation. (A) Measurement of IL-8 (pg/ml) by ELISA in HT29 cell supernatant after 16 h of incubation with 25% uninoculated BHIS (BHIS), 25% F. nucleatum BHIS conditioned medium <50-kDa fraction (<50 kDa), 25% F. nucleatum BHIS conditioned medium >50-kDa fraction (>50 kDa), or 5% purified F. nucleatum OMVs (OMVs) in DMEM in the absence or presence of TLR4 inhibitor CLI-095 (*n* = 6 replicates/experiment, repeated three independent times). (B) Measurement of TNF (pg/ml) by ELISA in HT29 supernatant after 16 h incubation with 25% uninoculated BHIS (BHIS), 25% F. nucleatum BHIS conditioned medium <50-kDa fraction, 25% F. nucleatum BHIS conditioned medium >50-kDa fraction, or 5% purified F. nucleatum OMVs (OMVs) in DMEM in the absence or presence of TLR4 inhibitor CLI-095 (*n* = 6 replicates/experiment, repeated three independent times). (C) Quantification of secreted luciferase in HT29 cells transfected with a pNFκB-MetLuc2-Reporter treated for 16 h (*n* = 9/experiment, repeated two independent times). (D) Western blot analysis of phosphorylated ERK, phosphorylated CREB, phosphorylated iκB, total iκB, and actin at 30 min incubation in HT29 cells (*n* = 3/experiment). Treatments are the same as in panels A, B, and C. Quantification of Western blots was performed using Fiji software. (E) Analysis of metabolic activity/viability in HT29 cells by resazurin assay (excitation, 560; emission, 600 nm). *, *P* < 0.05 (multi-way ANOVA).

OMVs from other Gram-negative species can activate innate immune responses, such as TLRs, which can activate the NF-κB pathway and drive proinflammatory cytokine responses (32). We hypothesized that F. nucleatum secreted virulence factors, such as OMVs, would promote proinflammatory effects in epithelial cells. To test this hypothesis, we cultured F. nucleatum subsp. *polymorphum* in BHIS (supplemented brain heart infusion medium) for 48 h and size-fractionated the supernatants to less than or greater than 50 kDa. The size-fractionated conditioned medium was applied to HT29 cell monolayers, and IL-8 production was measured to determine whether secreted factors from F. nucleatum stimulated a proinflammatory immune response. Conditioned medium fractions less than 50 kDa (<50 kDa) behaved similarly to the negative control (BHIS) and had no effect on IL-8 production by HT29 cells (Fig. 2A). However, the addition of conditioned medium fractions greater than 50 kDa (>50 kDa) containing particles above 2.4 nm, including OMVs, stimulated an ∼9-fold increase in IL-8 secretion compared to medium alone. That addition of purified F. nucleatum OMVs to HT29 cell monolayers also stimulated IL-8 production, suggesting that the active secreted factors in the >50-kDa fraction of conditioned media included OMVs. Pretreatment of HT29 cells for 1 h with the TLR4 inhibitor CLI-095 significantly attenuated the secretion of IL-8 in response to >50-kDa F. nucleatum conditioned media and OMVs. This result suggested that TLR4 activation results in stimulation of IL-8 production by F. nucleatum subsp. *polymorphum*. A similar pattern was observed for TNF secretion (Fig. 2B); >50-kDa and purified F. nucleatum OMVs stimulated an ∼6-fold increase in TNF secretion compared to uninoculated BHIS control and <50-kDa F. nucleatum conditioned media (Fig. 2B).

NF-κB is essential for upregulation of proinflammatory cytokines, including IL-8 (52). To assess whether NF-κB was activated by F. nucleatum secreted factors, we transfected HT29 monolayers with a pNFκB-MetLuc2-Reporter to monitor the activation of the NF-κB signal transduction pathway. Using this system, we observed a significant increase in secreted luciferase (indicating NF-κB activation) in response to the >50-kDa F. nucleatum conditioned media and purified OMVs compared to the medium control and <50-kDa F. nucleatum conditioned media (Fig. 2C). Incubation of HT29 cells with the TLR4 inhibitor resulted in an ∼2-fold decrease in NF-κB luciferase production. Next, we examined additional downstream targets TLR4, ERK, and CREB by Western blotting after incubating HT29 cells with fractionated conditioned media or purified OMVs for 30 min (Fig. 2D). As expected, media control and the <50-kDa F. nucleatum conditioned media did not activate p-ERK p-CREB or p-iκB at the 30-min time point. However, the addition of >50-kDa F. nucleatum conditioned media and purified OMVs increased the amounts of p-ERK, p-CREB, and p-iκB compared to media control. Importantly, we did not observe a decrease in cell viability/metabolism. In fact, we observed a slight increase in the conversion of resazurin to resorufin, suggesting an increase in cell metabolism in response to F. nucleatum conditioned media (Fig. 2E). These data demonstrate a robust response of colonic epithelial cells to factors secreted by F. nucleatum subsp. *polymorphum*.

**FIG 3:**
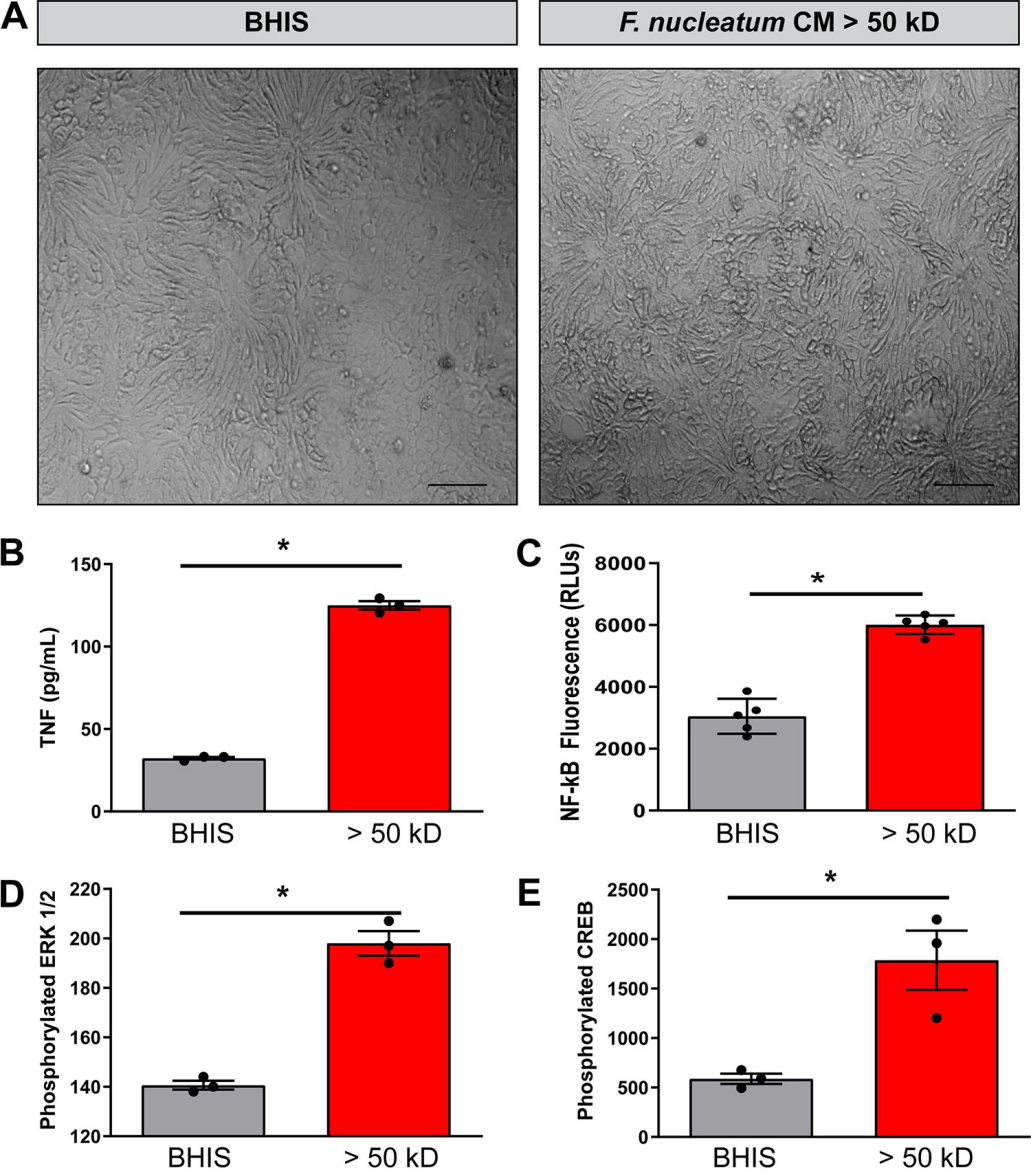
F. nucleatum >50-kDa compounds promote TNF, NF-κB, and MAPK signaling in human colonoid monolayers. (A) Representative images of human colonoid monolayers treated with 25% BHIS (BHIS) or 25% F. nucleatum conditioned medium >50-kDa fraction (>50 kDa) in DMEM, 1× HEPES, 1× GlutaMAX, and pyruvate for 16 h (scale bar,100 μm). (B) Measurement of TNF (pg/ml) by ELISA in colonoid monolayers treated with 25% BHIS (BHIS) or 25% F. nucleatum BHIS conditioned medium >50-kDa fraction after 16 h incubation (*n* = 4 monolayers/experiment, repeated two independent times). (C) Quantification of secreted luciferase in human colonoid monolayer cells transfected with a pNFκB-MetLuc2-Reporter treated for 16 h (*n* = 4 monolayers/experiment). (D and E) Luminex Magpix multiplex analysis of phosphorylated ERK (D) and CREB (E) in human colonoid monolayers treated with 25% BHIS (BHIS) or 25% F. nucleatum conditioned medium >50-kDa fraction for 1 h (*n* = 3 monolayers/experiment). *, *P* < 0.05 (Student *t* test).

While HT29 colonic cancer-derived cells can model some intestinal epithelial functions, they do not reflect the intestinal epithelium as a whole (53). The human intestinal enteroid (HIE; also known as organoid) system has expanded our *in vitro* capabilities in understanding the physiology of the noncancerous human intestinal epithelium. HIEs are derived from intestinal stems cells and provide a long-term primary culture system. Importantly, HIEs harbor all cell lineages found in native tissue, are segment specific, and contain TLRs (53–55). We have previously shown that HIE media contains a number of antioxidants, including *N*-acetylcysteine, glutathione, B27 supplement, and N2 supplement, which dampen proinflammatory signaling cascades (54). However, by using a simplified media without anti-oxidants, we can generate HIEs that are responsive to microbial stimulation such as lipopolysaccharides (LPS), lipoteichoic acid, and flagellin. We used HIEs derived from colonic epithelial stem cells isolated from healthy adults to examine the effects of F. nucleatum secreted compounds on the uninflamed intestinal epithelium. Observation by light microscopy showed that treatment of colonic HIE monolayers with >50-kDa F. nucleatum conditioned media did not affect cell morphology (Fig. 3A). Treatment with the >50-kDa F. nucleatum conditioned media promoted TNF secretion by colonic HIE monolayers compared to the media control (Fig. 3B). In contrast to our HT29 model, we found no differences in IL-8 secretion between media control and F. nucleatum conditioned media (data not shown). Transfection of colonic HIE monolayers with the pNFκB-MetLuc2-Reporter confirmed upregulation of NF-κB after treatment with F. nucleatum conditioned medium (Fig. 3C). Analysis of HIE cell lysates by Luminex Magpix revealed increased p-ERK and p-CREB after treatment with >50-kDa F. nucleatum conditioned media, consistent with our HT29 cell data. These data confirm our HT29 cell data and demonstrate that >50-kDa compounds produced by F. nucleatum can stimulate epithelial inflammatory signals.

### *F. nucleatum* subsp. *polymorphum* promotes inflammation in a humanized mouse model following antibiotic administration.

**FIG 4:**
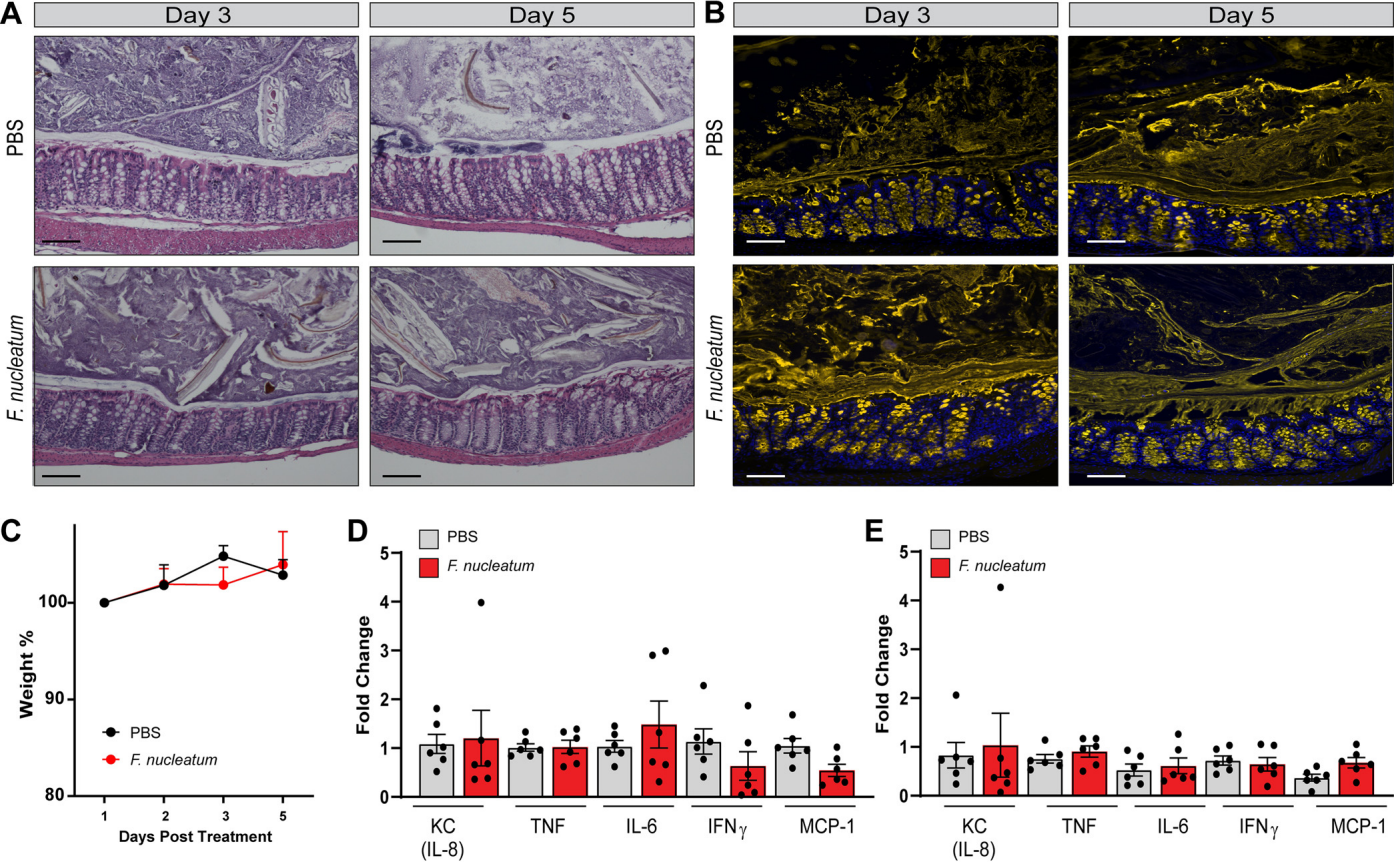
F. nucleatum subsp. *polymorphum* is unable to promote inflammation in the presence of a complete gut microbiome. (A) Representative images of H&E stains of control animals and F. nucleatum subsp. *polymorphum*-treated animals at day 3 and day 5 postinfection (scale bar, 100 μm). (B) FISH staining of *Fusobacterium* (red) counterstained with MUC2 (yellow) and Hoechst (blue) at day 3 and day 5 postinfection (scale bar, 100 μm). (C) Analysis of mouse weights at days 1, 2, 3, and 5 postinfection (*n* = 6/group). *, *P* < 0.05 (repeated-measures ANOVA). (D) Colonic mRNA expression of proinflammatory related genes on day 3 postinfection (*n* = 6/group). *, *P* < 0.05 (two-way ANOVA). (E) Colonic mRNA expression of proinflammatory related genes on day 5 postinfection (*n* = 6/group). *, *P* < 0.05 (two-way ANOVA).

Based on our promising *in vitro* data, we next addressed whether F. nucleatum could elicit proinflammatory responses using a mouse model (Fig. 4). Since *Fusobacterium* spp. are commonly found in the gastrointestinal tracts of humans, but not mice (56), and may have unique interactions with human-derived microbes, we used mice colonized with a human intestinal microbiota, also known as humanized microbiota mice. Mice were orally gavaged with a single dose of F. nucleatum (10^9^ CFU) and euthanized on day 3 and day 5 postinoculation with F. nucleatum. No changes were observed in the crypt architecture or immune infiltration of the intestinal epithelium from mice treated with F. nucleatum at days 3 or 5 postinoculation (Fig. 4A). Likewise, F. nucleatum was not identified in the colonic mucus layer by FISH (Fig. 4B), although low levels of *Fusobacterium* gDNA was found in the feces by quantitative PCR (qPCR) analysis (Table 1), and no weight differences were observed between groups (Fig. 4C). A closer examination of colonic gene expression revealed no changes in the proinflammatory cytokine gene expression of KC (the mouse homolog to IL-8), IL-6, IFN-γ, and monocyte chemoattractant protein-1 (MCP-1) in F. nucleatum-treated mice at days 3 or 5 when an intact gut microbiota was present (Fig. 4D and E). These findings suggest that F. nucleatum does not have detrimental effects on overall health parameters in the setting of an intact human microbiota.

**TABLE 1:** Calculated *Fusobacterium* fecal load based on standard cultures of F. nucleatum subspecies *polymorphum*

Treatment	Mean *Fusobacterium* CFU ± SEM^*a*^	
	No Abx	Abx
Day 3		
PBS	0	0
*F. nucleatum*	3.1 × 10^1^ ± 0.6 × 10^1^	2.4 × 10^4^ ± 1.5 × 10^3^
		
Day 5		
PBS	0	0
*F. nucleatum*	0	4.3 × 10^3^ ± 0.8 × 10^3^

a As determined by qPCR. Abx, antibiotics.

**FIG 5:**
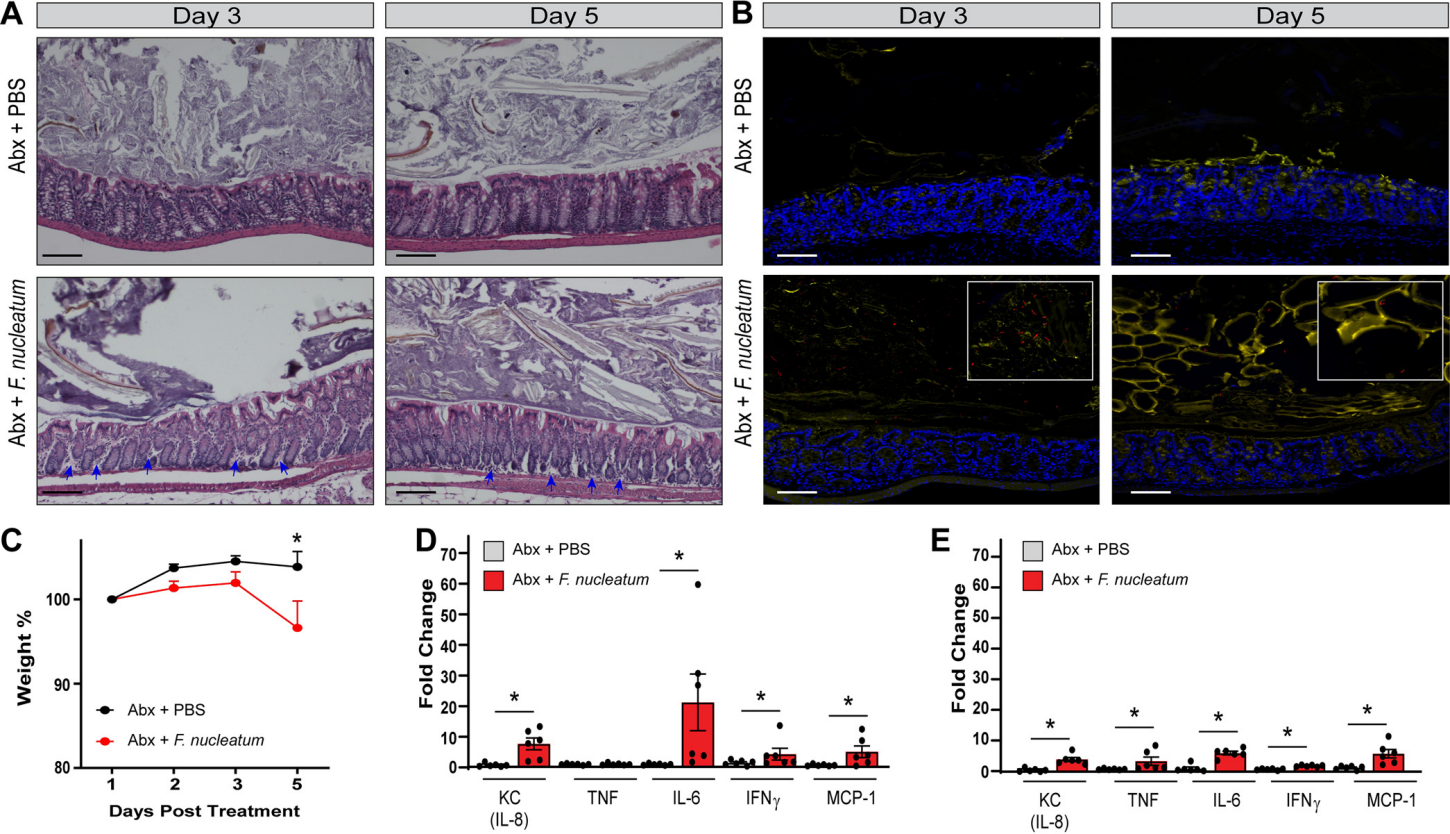
F. nucleatum subsp. *polymorphum* drives inflammation *in vivo* with antibiotic disruption of the gut microbiome. (A) Representative images of H&E stains of control animals and F. nucleatum subsp. *polymorphum*-treated animals who received antibiotics at day 3 and day 5 postinfection (scale bar, 100 μm). Blue arrows highlight immune infiltration. (B) FISH staining of *Fusobacterium* (red) counterstained with MUC2 (yellow) and Hoechst (blue) at day 3 and day 5 postinfection. Enlarged insets demonstrate *Fusobacterium* at day 3 and 5 (scale bar, 100 μm). (C) Analysis of mouse weights at days 1, 2, 3, and 5 postinfection (*n* = 6/group). *, *P* < 0.05 (repeated-measures ANOVA). (D) Colonic mRNA expression of proinflammatory related genes on day 3 postinfection (*n* = 6/group). *, *P* < 0.05 (two-way ANOVA). (E) Colonic mRNA expression of proinflammatory related genes on day 5 postinfection (*n* = 6/group). *, *P* < 0.05 (two-way ANOVA).

We previously detected increased *Fusobacterium* operational taxonomic unit (OTU) abundance in stool samples from patients on antibiotics (57). As result, we reasoned that F. nucleatum may require an available niche to promote intestinal inflammation. To address this question, humanized microbiota mice were treated with a cocktail of antibiotics (kanamycin, gentamicin, colistin, metronidazole, and vancomycin) for 5 days, followed by a single injection of clindamycin. This broad-spectrum antibiotic regimen has previously been shown decrease multiple bacterial OTUs by 16S rRNA sequencing (58). Directly after antibiotic treatment, the mice were orally gavaged with F. nucleatum (10^9^ CFU). This treatment regimen was designed to alter the microbiome and provide a potential niche for F. nucleatum. The intestinal epithelium from mice euthanized at day 3 postinoculation with F. nucleatum exhibited disruption of the colonic architecture, with increased immune infiltration and a depleted mucus layer which resulted in luminal contents being closer in proximity to the intestinal epithelium (Fig. 5A). After 5 days postinoculation with F. nucleatum, colonic epithelia of mice exhibited reduced architecture disruption and immune infiltration compared to day 3, but still displayed loss of goblet cells and a thinner mucus layer. Fluorescence *in situ* hybridization (FISH) confirmed the presence of F. nucleatum in the epithelial mucus layer at both days 3 and 5, with the greatest numbers of bacteria observed at day 3 (Fig. 5B and Table 1). Oral gavage with F. nucleatum also correlated with weight loss compared to phosphate-buffered saline (PBS)-treated mice, supporting the notion that F. nucleatum had negative effects on health (Fig. 5C). Analysis of colonic gene expression revealed increased concentrations of epithelial- and immune-cell-secreted KC (the mouse IL-8 homologue) and immune-cell-secreted IL-6, IFN-γ, and MCP-1 in F. nucleatum-treated mice compared to PBS treatment at day 3 (Fig. 5D). IL-6 showed the greatest change in increased expression at day 3 postinoculation with F. nucleatum. With the exception of TNF, cytokine gene expression was substantially lower in the F. nucleatum group on day 5 compared to that observed on day 3 (Fig. 5E). However, KC, TNF, IL-6, IFN-γ, and MCP-1 were still increased in the F. nucleatum-treated mice compared to mice gavaged with PBS control. These data indicate that F. nucleatum is capable of driving a proinflammatory signaling cascade *in vivo* in the presence of an antibiotic-disrupted humanized microbiota. Collectively, these findings expand our knowledge of F. nucleatum-host interactions and indicates that orally derived F. nucleatum can stimulate inflammatory responses.

## DISCUSSION

Providing a deeper understanding of how F. nucleatum promotes inflammation could potentially lead to novel therapeutic approaches for the treatment of multiple intestinal diseases. Our data indicate that F. nucleatum subsp. *polymorphum* secretes OMVs, which can activate TLR4 and downstream targets ERK, CREB, and NF-κB, thereby promoting proinflammatory cytokine production. These effects were observed in colonic HT29 cells, as well as in human colonoid (organoid) monolayers. These *in vitro* data support the hypothesis that F. nucleatum is capable of eliciting intestinal inflammation through the production of secreted compounds, among other mechanisms. In mice harboring a human microbiome, we found that antibiotic treatment allowed F. nucleatum to adhere to the intestinal mucus layer and drive inflammation, as indicated by weight loss, increased immune infiltration, altered colonic architecture, and proinflammatory cytokine mRNA signatures. We also found that antibiotic-mediated depletion of the gut microbiome is essential for F. nucleatum-mediated effects. These data provide solid evidence that F. nucleatum can promote inflammation in the gastrointestinal tract when an open niche is available.

The majority of research on F. nucleatum has focused on its role as a periodontal pathogen. However, in recent years investigators have begun to view F. nucleatum as an intestinal pathogen as well (11, 12). This is largely due to the identification of F. nucleatum in colonic biopsy specimens from patients with IBD and colorectal cancer (10, 11, 13, 14, 17, 29, 30, 35, 59–66). Many Gram-negative bacteria, including F. nucleatum, release OMVs both *in vitro* and *in vivo* (67–69), and these nanoparticles have been implicated as major players in bacterial pathogenesis. OMVs commonly contain LPS, DNA, adhesins, and enzymes and therefore have been proposed to act as a delivery system for these virulence factors (70). Our *in vitro* work indicates the TLR4 activation by F. nucleatum-conditioned media, including OMVs, play a significant role in epithelial cytokine production. As a result, we speculate that outer membrane LPS may be driving this effect. Consistent with this hypothesis, we observed that application of purified LPS from F. nucleatum subsp. *polymorphum* also stimulated IL-8 in our HT29 cells (data not shown). However, we do not think that TLR4 is the only pathway employed by F. nucleatum secreted compounds and OMVs. In colon cancer studies, F. nucleatum stimulation of proinflammatory cytokines was found to occur by both TLR4-dependent and -independent mechanisms (71). Park et al. demonstrated that F. nucleatum activates both TLR2 and TLR4 in bone-marrow-derived macrophages to stimulate IL-6 production (72), an effect that is completely ablated in the absence of MyD88. Thus, it is likely that other TLRs may be activated in response to F. nucleatum-secreted products. As a result, we speculate that OMVs activate epithelial cells and immune cells through both TLR4-dependent and -independent mechanisms *in vivo*. We speculate that, similar to cancer models, inflammation associated with F. nucleatum is likely dependent on MyD88 signaling. In addition to LPS, there are many proteins in F. nucleatum OMVs with potential virulence functions. These proteins include FomA, FadA, FadD, Fad-I, NapA, ClpB, GroEL, TraT, and YadA; future studies on their contributions to disease are needed.

In addition to OMVs, F. nucleatum may secrete other compounds which stimulate TLRs and drive inflammation. Although our *in vitro* studies suggest that OMVs contribute to inflammation, it is possible that our >50-kDa fraction also contains other large-molecular-weight compounds capable of stimulating cytokines. In addition, it is possible that large-molecular-weight compounds act in synergy with OMVs to drive inflammation. Our *in vivo* studies do not exclude other mechanisms of inflammation. Future studies are warranted to address the role of other factors in stimulating inflammatory signals.

In addition to secreted factors, several studies have found that F. nucleatum is capable of invading epithelial cells and can directly activate proinflammatory signals (73–75). F. nucleatum invasion of oral epithelial cells activates NF-κB and induces proinflammatory cytokines (IL-8, TNF, IL-1β, and IL-6) (73–75). In the setting of cancer, F. nucleatum invasion of cancer cells also induces NF-κB and proinflammatory cytokine production (11, 29, 30, 35, 62, 64, 71). Although we observed cytokine production and inflammation in our studies, we saw little evidence of epithelial invasion by F. nucleatum by our FISH staining. We found that F. nucleatum can adhere to colonic mucin glycans and predict mucus adhesion may limit the invasion of F. nucleatum into the epithelium. Another possible explanation of the lack of invasion may be explained by the intact epithelium in our mouse model. It is possible that F. nucleatum may require damage or epithelial fragility to invade the colonic epithelium. In addition, our findings may be strain dependent, since clinical isolates from patients with IBD have been characterized to be more proinflammatory than F. nucleatum isolated from healthy subjects. For example, F. nucleatum isolated from inflamed regions of the gut exhibit enhanced invasion of Caco-2 cells and trigger TNF (76). These results suggest that although F. nucleatum may be proinflammatory in general, some strains are more pathogenic than others.

Our data indicate that F. nucleatum requires disruption of the microbiome to promote inflammation. Previous work from Collins et al. using the same humanized mouse model demonstrated that antibiotic treatment significantly reduced the levels of *Lachnospiraceae*, *Bacteroidaceae*, *Clostridiaceae*, and *Verrucomicrobiaceae* compared to mice without antibiotics (58). These findings appear to resemble that of microbial composition in intestinal microbiomes of patients in IBD, both ulcerative colitis (UC) and Crohn’s disease (CD), whereas a diminution of *Lachnospiraceae* and *Bacteroidetes* has been observed compared to healthy volunteers or non-IBD controls (77–80). In addition, the gut microbiota of the patients with colorectal cancer are often depleted in *Lachnospiraceae*, *Bacteroidetes*, and *Clostridia* and enriched in *Fusobacterium* (65, 81–85). One study identified that high abundance of *Lachnospiraceae* was negatively associated with the colonization of colonic tissue by oral microbes (*Fusobacterium*, *Streptococcus*, *Gemella*, etc.) (84). These microbiome studies suggest a protective colonization resistance role for select gut microbes, such as *Lachnospiraceae* and commensal *Bacteroides.* We theorize that the presence of *Lachnospiraceae*, *Bacteroidetes*, and other antibiotic-depleted microbes may prevent F. nucleatum colonization and therefore inflammation.

The intestinal microbiome is resilient and can revert back toward the original population following antibiotic treatment. Consistent with this notion, Collins et al. observed resolution of the microbiome in the same human microbiome mouse model following antibiotics and predicted that the microbial communities would eventually return to baseline (58). As a result, we speculate that F. nucleatum subsp. *polymorphum* would not persist in our antibiotic-treated mouse model long term. We predict that as the microbiome returned, F. nucleatum would be outcompeted, and there would be resolution of inflammation. By day 5 postgavage, we observed less F. nucleatum by FISH staining and lower inflammatory markers in the antibiotic-treated mice compared to day 3. We predict that the effects of F. nucleatum would only remain for a few more days (ca. days 7 to 10) as the microbiome returned to its usual complexity. To fully address this question, more studies are needed to determine the precise balance of F. nucleatum and the microbiome following antibiotic administration.

*Fusobacterium* is commonly found in mixed microbial infections (86). This is due in part to the communal nature of F. nucleatum. It harbors multiple adhesins which promote multispecies biofilm formation (87–92). These microbe-microbe interactions have been well documented in the oral cavity; however, biofilm formation may also be a potential strategy for F. nucleatum colonization in the gut. It is possible that the microbes present after antibiotics interact with F. nucleatum and promote its persistence. Since oral microbes are commonly found in intestinal disease states and F. nucleatum is known to aggregate and form biofilms with multiple oral bacteria (87–91, 93–97), a synergy may exist between these groups to promote intestinal inflammation and pathology. Ledder et al. demonstrated that F. nucleatum can coaggregate with intestinal microbes, including Bifidobacterium adolescentis and Lactobacillus paracasei, and to a lesser degree with Bacteroides vulgatus and Enterococcus faecium (91). Therefore, F. nucleatum may be interacting with mucosa-associated gut microbes to enhance colonization.

**FIG 6:**
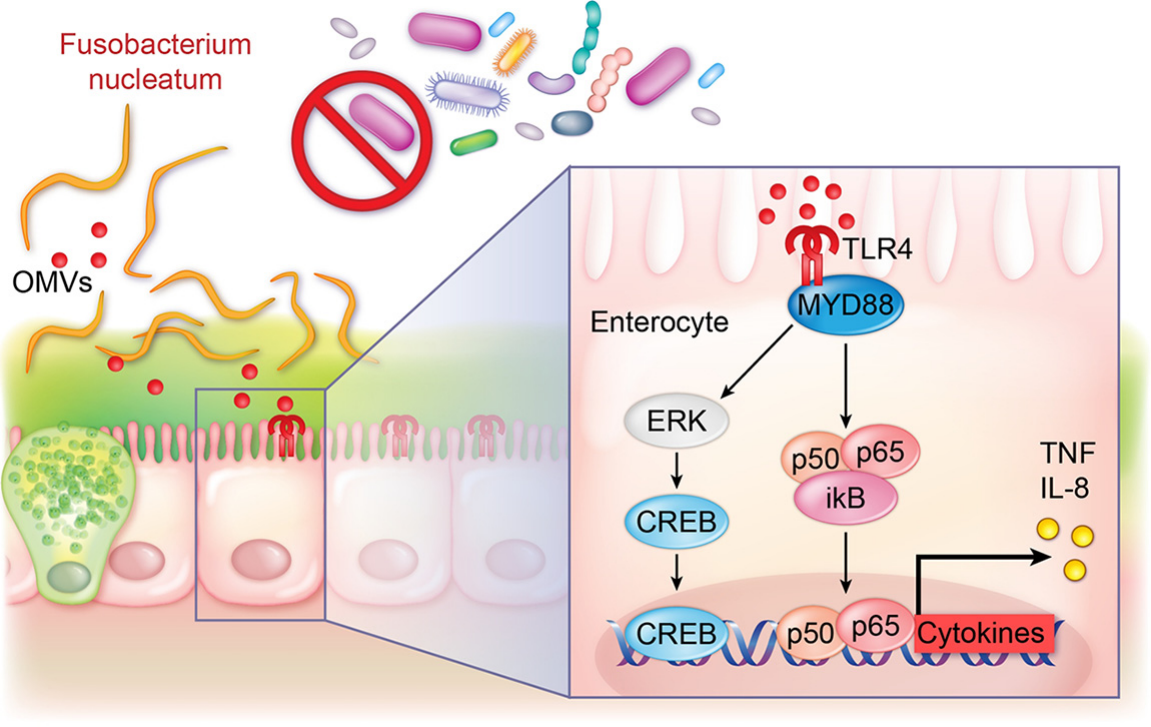
Proposed model for F. nucleatum subsp. *polymorphum*-driven inflammation. F. nucleatum adheres the intestinal mucus layer in the setting of an altered gut microbiome, in which it delivers secreted compounds as “cargo” in OMVs. The OMVs activate epithelial TLRs, including TLR4, which promotes phosphorylation and activation of ERK, CREB, and NF-κB, thereby driving the production of proinflammatory cytokines and initiating inflammation.

Overall, our findings indicate that F. nucleatum can promote inflammation in normal epithelial cells *in vitro* and *in vivo* (Fig. 6). We speculate that certain strains of F. nucleatum in genetically susceptible patients may be an initiating or contributing factor to inflammation. We predict that in patients undergoing antibiotics, the microbiome does not provide colonization resistance and F. nucleatum can establish residence. We also reason that an aberrant immune response coupled with an altered microbiome and F. nucleatum could lead to chronic inflammation. As a result, our findings point to F. nucleatum and OMVs as drivers of intestinal inflammation and warrant further study as future targets for treatment strategies aimed at reducing mucosal inflammation.

## MATERIALS AND METHODS

### Bacterial culture conditions.

Fusobacterium nucleatum subsp. *polymorphum* ATCC 10953 (American Type Culture Collection) was cultured in brain heart infusion medium (Difco) supplemented with 2% yeast extract and 0.2% cysteine (BHIS) anaerobically at 37°C in an anaerobic workstation (Anaerobe Systems AS-580) with a mixture of 5% CO_2_, 5% H_2_, and 90% N_2_.

**(i) Preparation of *F. nucleatum* subsp. *polymorphum* conditioned media.** To assay compounds secreted by F. nucleatum, we prepared conditioned media as follows. Overnight cultures were subcultured into BHIS at an optical density at 600 nm (OD_600_) of 0.1 and cultured anaerobically for 48 h at 37°C. Cells were centrifuged at 7,000 × *g* for 5 min, and the supernatant was filter sterilized using 0.45-μm polyvinylidene difluoride Millipore centrifuge filters. This filtered supernatant was termed conditioned media (CM). F. nucleatum CM was size fractionated with Amicon Ultra 50-kDa centrifugal filters (Millipore, UFC905024). The fraction below 50 kDa was termed “<50 kDa” and the fraction above 50 kDa was termed “>50 kDa.”

**(ii) Isolation of *F. nucleatum* subsp. *polymorphum* outer membrane vesicles.** We isolated OMVs produced by F. nucleatum subsp. *polymorphum* as follows. BHIS medium (500 ml) was inoculated to an OD_600_ of 0.1 using F. nucleatum overnight cultures and incubated anaerobically at 37°C for 48 h. Cells were pelleted by centrifugation at 7,000 × *g* for 10 min. Cell-free supernatant was collected, mixed with 120 g of ammonium sulfate, and then incubated for 2 h at 4°C. The vesicle-containing precipitate was collected by centrifugation at 10,000 × *g* for 20 min, and the pellets resuspended in 50 mM HEPES buffer (pH 7.5). The resuspended pellets were dialyzed overnight in 50 mM HEPES (pH 7.5) buffer at 4°C using 10,000-molecular-weight cutoff dialysis tubing. The vesicles were concentrated with >50-kDa Amicon Ultra centrifugation filters and added to OptiPrep solution (Sigma, D1556) at a ratio of 1:3 (vol/vol). This suspension was then added to 45% OptiPrep in Ultraclear centrifuge tubes and ultracentrifuged at 292,700 × *g* (70 Ti rotor; Beckman Coulter Inc.) for 3 h. Sequential fractions were collected and analyzed by 15% SDS-PAGE to identify fractions containing vesicles. Vesicle-containing fractions were pooled, diluted 10-fold in DPBS (Thermo Fisher, catalog no. 14190144), and separated from the OptiPrep by centrifugation at 38,400 × *g* for 3 h to remove the OptiPrep solution. Finally, the purified OMVs were resuspended in 500 μl of DPBS and used for microscopy and tissue culture experiments.

**(iii) OMV analysis measured by NTA.** Nanoparticle tracking analysis (NTA) was performed to determine the size of the OMVs using a NanoSight LM10 instrument (Malvern, Westborough, MA). Equipped with a sample chamber, a laser light source of 532 nm, sCMOS camera, and an optical microscope. The samples were diluted by 200-fold in Millipore water and the particle concentration were between 1.2E+8 to 4.0E+8 particles/ml. The samples were injected into the LM10 unit with a 1-ml syringe at a syringe pump speed of 100. The capturing settings (camera level, slider shutter, and gain) were adjusted automatically. The NanoSight LM10 recorded 30-s sample videos, which were analyzed by using NTA3.4 software. The particle diameter was obtained from the Stokes-Einstein equation.

**(iv) Fluorescent labeling of *F. nucleatum* subsp. *polymorphum*.** Fluorescently tagged F. nucleatum subsp. *polymorphum* were generated by incubation with 10 μM carboxyfluorescein diacetate succinimidyl ester (CFDA-SE) as previously described (98). Briefly, F. nucleatum subsp. *polymorphum* was grown overnight in BHIS anaerobically at 37°C. The following day, the cultures were centrifuged at 5,000 × *g* for 5 min, and the pellet was washed twice with anaerobic PBS. F. nucleatum was then incubated with 10 μM CFDA-SE (Thermo Fisher, C1157) in PBS anaerobically at 37°C for 1 h. After incubation, CFDA-SE tagged cultures were centrifuged at 5,000 × *g* for 5 min, and the pellet was washed three times with anaerobic PBS to remove the residual CFDA-SE. Fluorescence was confirmed by microscopy.

### Tissue culture.

*In vitro* experiments were performed with the human colon cell line HT-29 (ATCC HTB-38). HT29 cells were maintained in McCoy’s 5A medium (ATCC) supplemented with heat-inactivated 10% fetal bovine serum (FBS; Invitrogen) and antibiotics (100 U/ml penicillin and 100 μg/ml streptomycin) at 37°C and 5% CO_2_. For adhesion assays, the mucin-producing human colon T84 line (ATCC CL-248) was used. T84 cells were grown in Dulbecco modified Eagle medium (DMEM; Thermo Fisher) supplemented with 10% FBS in a humidified atmosphere at 37°C and 5% CO_2_. Purified MUC2 was isolated from T84 cells and adhered to glass coverslips as previously described (99). For monolayer adhesion, T84 cells were seeded at 5 × 10^5^ cells/well in a 24-well plate containing poly-l-lysine-coated glass coverslips and grown to confluence. Prior to adhesion assays, T84-coated coverslips were incubated with Hoechst 33342 (Invitrogen, H3570) for 10 min at room temperature to stain the epithelial nuclei. Fluorescently tagged F. nucleatum subsp. *polymorphum* was added to either T84 monolayers or APTS-coated MUC2-coated coverslips and incubated for 1 h at 37°C and 5% CO_2_. After incubation, coverslips were washed three times with PBS and fixed with Clarke’s fixative to preserve the mucus layer, and mounted coverslips were examined by microscopy.

To examine cytokine production, HT29 cells were seeded at 1 × 10^4^ cells/well in 96-well tissue culture-treated plates (Corning) and incubated at 37°C and 5% CO_2_ overnight. The following day, the cells were treated with either DMEM (without FBS or media), 25% uninoculated BHIS media in DMEM (BHIS), 25% F. nucleatum conditioned medium (<50-kDa fraction) in DMEM, 25% F. nucleatum conditioned medium (>50-kDa fraction) in DMEM, or 5% F. nucleatum OMVs in DMEM and then incubated overnight at 37°C and 5% CO_2_. To examine the contribution of TLR4 to cytokine production, HT29 cells in 96-well plates were pretreated for 1 h with 1 μM CLI-095 (TLR4 inhibitor; InvivoGen, TLRLCLI95) and maintained in 1 μM CLI-095 throughout an overnight incubation. Supernatants were examined for IL-8 production by IL-8/CXCL8 DuoSet ELISA (R&D Systems, DY208-05) and TNF production by TNF-α DuoSet ELISA (R&D Systems, DY210-05). Cell viability/metabolism was confirmed by using the dye resazurin (7-hydroxy-3H-phenoxazin-3-one 10-oxide; Sigma, R7017) at a final concentration of 44 μM. Cells were incubated for 3 h at 37°C and 5% CO_2_, and the fluorescence resulting from resazurin reduction to resorufin was analyzed using a microplate spectrofluorometer at an excitation wavelength of 570 nm and an emission wavelength of 600 nm.

For Western blot analysis, HT29 cells were seeded at 2 × 10^5^ cells/well in 24-well tissue culture treated plates (Corning). After growing cells to confluence, the monolayers were treated as described for the 96-well plate assay (DMEM, 25% BHIS, 25% <50 kDa, 25% >50 kDa, and 5% OMVs) in DMEM (no FBS) for 30 min. Cells were then lysed with radioimmunoprecipitation assay buffer (Thermo Fisher Scientific, Waltham, MA) containing a protease inhibitor cocktail (Roche). After centrifugation, the protein concentrations were quantitated by a Bradford assay (100). Portions (50 μg) of total protein were resolved by SDS-PAGE and transferred to polyvinylidene difluoride (PVDF) membranes (Millipore, Billerica, MA). After a blocking step with 5% milk in PBS-Tween 20 (PBS-T) for 30 min at room temperature, the PVDF membranes were incubated at 4°C overnight with antibodies for phosphorylated ERK, CREB, and iκBα, as well as total iκBα and β-actin. After three washes with TBS-T, the PVDF membranes were incubated with horseradish peroxidase-conjugated secondary antibodies for 1 h at room temperature. After TBS-T washes, the membranes were developed with ECL substrate (GE Healthcare, Buckinghamshire, UK). Western blots were analyzed using Fiji (formerly ImageJ) software (National Institutes of Health).

To examine NF-κB activation, HT29 monolayers were grown to 75% confluence and transiently transfected with a NF-κB secreted luciferase reporter (Clontech, pNFκB-MetLuc2-Reporter) in Opti-MEM (Thermo Fisher) using the XtremeGene HP DNA transfection reagent (Roche) (101) at a final concentration of 0.6 μl of XtremeGene HP and 0.3 μg of DNA per well. HT29 monolayers were incubated for 48 h at 37°C and 5% CO_2_. After transfection, the cells were treated with DMEM, 25% BHIS, 25% <50 kDa, 25% >50 kDa, and 5% OMVs) in DMEM (no FBS) for 16 h. The supernatants were examined for luciferase activity using a Lonza Lucetta tube luminometer.

### Human colonoid cultures.

The human stem cell-derived colonoid line C103 was purchased from the Baylor College of Medicine GEMs enteroid core. Three-dimensional human colonoids were cultured in complete medium with growth factors (CMGF+) in phenol red-free, growth factor-reduced Matrigel (Corning) as previously described (102–104). Colonoids at passage 9 were seeded into flat 96-well plates as described previously (104–109). Briefly, three-dimensional colonoids were dislodged from Matrigel domes, washed with an ice-cold solution of 0.5 mM EDTA in 1× PBS, and dissociated at 37°C for 4 min with 0.05% trypsin and 0.5 mM EDTA. After 4 min, the trypsin was inactivated with Advanced DMEM/F-12, 1× GlutaMAX, and 1× HEPES continuing 10% FBS. The dissociated colonoids were filtered through a 40-μm nylon cell strainer (Falcon, catalog no. 352340) to generate single cells and then suspended with CMGF+ and 10 μM Y-27632 Rock inhibitor. The solution was added to Matrigel-precoated 96-well plates, followed by incubation for 48 h at 37°C and 5% CO_2_. After 48 h, the medium was changed to differentiation medium, which contains the same components as CMGF+ but without Wnt3A conditioned medium, R-spondin conditioned medium, SB202190, and nicotinamide and only 5% (vol/vol) Noggin conditioned medium, but was still supplemented with 10 μM Y-27632 Rock inhibitor. The differentiation medium was changed daily for 5 days.

To examine F. nucleatum stimulation of colonoid monolayers, the differentiation medium was changed to DMEM supplemented with 1× HEPES, 1× GlutaMAX, and 1× pyruvate. This simplified media has previously been demonstrated to improve cytokine production by human colonoids (54). Colonoids were treated with either 25% uninoculated BHIS or 25% F. nucleatum conditioned media (>50 kDa) in DMEM/HEPES/GlutaMAX/pyruvate media. For cytokine analysis, colonoids were treated for 16 h, and the supernatants were examined for IL-8 and TNF by ELISA. In order to examine NF-κB activation, 96-well colonoid monolayers were transduced on day 3 with NF-κB secreted luciferase reporter (Clontech, pNFκB-MetLuc2-Reporter) and incubated for an additional 2 days in differentiation media. After 16 h incubation with either 25% uninoculated BHIS or 25% >50 kDa, the supernatants were examined for secreted luciferase as described above. For intracellular signaling analysis, colonoid monolayers were treated for 1 h, washed with PBS containing Ca^2+^ and Mg^2+^, lysed with Luminex lysis buffer, and analyzed with a Milliplex MAP multi-pathway total magnetic bead assay (Millipore, catalog no. 48-681-MAG) with a Magpix instrument (Luminex Corporation, Austin, TX). Magpix analysis was performed by the Functional Genomics and Microbiome Core of the Texas Medical Center Digestive Diseases Center. Data were collected and analyzed by using Luminex xPONENT for MAGPIX, version 4.2, build 1324, and Milliplex Analyst version 5.1.0.0, standard build 10/27/2012.

### Animal models.

Animal experiments were approved by the Institutional Animal Care and Use Committee (IACUC) at Baylor College of Medicine. For animal experiments, F. nucleatum subsp. *polymorphum* was cultured overnight anaerobically in BHIS and centrifuged at 7,000 × *g* for 5 min. The bacterial pellet was washed twice, with sterile anaerobic PBS viability confirmed by serial plating F. nucleatum on BHIS agar to calculate the CFU; the cells were adjusted to 10^9^ cells ml^−1^ and used to treat animals as described below. Humanized microbiota C57BL/6 mice were generated as described previously (58) and maintained in a BCM BSL-2-approved animal facility. Adult mice (10 to 16 weeks) were administered an antibiotic cocktail (kanamycin [0.4 mg ml^−1^], gentamicin [0.035 mg ml^−1^], colistin [850 U ml^−1^], metronidazole [0.215 mg ml^−1^], and vancomycin [0.045 mg ml^−1^]) *ad libitum* in drinking water for 3 to 5 days as previously described (58). After 24 h, the mice were treated with clindamycin (10 mg kg^−1^, injected intraperitoneally). Mice were gavaged orally with sterile PBS (control) or F. nucleatum subsp. *polymorphum* in PBS (10^9^ CFU) 24 h later. Mice were monitored twice daily and euthanized on day 3 and day 5 after oral gavage. No visual or behavioral differences were noted in mice receiving antibiotics compared to control mice (no antibiotic). To examine the contribution of the microbiome on F. nucleatum*-*induced inflammation, a subset of mice did not receive any antibiotic treatment and only received PBS (control) or F. nucleatum subsp. *polymorphum* in PBS (10^9^ CFU). For all experiments, groups contained equal numbers of male and female mice to exclude sex bias (6 females/6 males per treatment group).

### Intestinal tissue staining.

**(i) H&E and PAS-AB.** Mouse colons were placed intact in cassettes and fixed in 10% Carnoy’s fixative. Paraffin-embedded tissue sections (7 μm) were processed for hematoxylin and eosin (H&E) or periodic acid-Schiff/Alcian blue (PAS-AB) staining. H&E and PAS-AB sections were examined by bright-field and imaged on the Nikon Eclipse 90i (Nikon) microscope using a DS-Fi1-U2 camera (Nikon) with a differential interference contrast (DIC) objective.

**(ii) Immunofluorescence.** F. nucleatum localization was examined using a *Fusobacterium*-specific FISH probe (5′-CGCAATACAGAGTTGAGCCCTGC-3′), and total bacteria were examined using a universal bacterial FISH probe EUB338 (5′-GCTGCCTCCCGTAGGAGT-3′; Integrated DNA Technologies [IDT]) (110). Briefly, tissue sections were dehydrated and incubated with the *Fusobacterium* probe at 45°C in a dark humidifying chamber, hybridized for 45 min, and counterstained with MUC2 (1:200 dilution; Cloud-Clone Corp., PAA705Mu01) and Hoechst 33342 (Invitrogen, H3570). Immunostained slides were imaged on an Eclipse 90i (Nikon, Tokyo, Japan) with a 20× Plan Apo (NA 0.75) DIC objective, and the images were recorded using a CoolSNAP HQ2 camera (Photometrics) using a Nikon Intensilight C-HGFI mercury lamp.

### Transmission electron microscopy of OMVs.

F. nucleatum and OMVs were prepared for transmission electron microscopy (TEM) by fixing in 2.5% glutaraldehyde in 0.1 M cacodylate buffer at room temperature for 1 h, followed by further fixation at 4°C. Samples were postfixed in 1% tannic acid for 1 h, followed by 1% osmium tetroxide for 1 h and en bloc stained with 1% uranyl acetate. The samples were dehydrated with a graded ethanol series. Samples were infiltrated into Quetol-Spurrs resin using propylene oxide as a transition solvent and polymerized at 60°C for 48 h, as previously described (111). The resulting blocks were sectioned at 70 nm on 300-mesh copper grids and imaged on a Tecnai T12 transmission electron microscope at 100 kV using an AMT CMOS camera. OMV sizes were measured using Fiji software (NIH) from TEM images.

### RNA isolation, gDNA isolation, and qPCR.

RNA was extracted from mouse colons using TRIzol according to manufacturer details (Thermo Fisher, catalog no. 15596018). RNA (1 μg) was converted to cDNA using the SensiFAST cDNA synthesis kit (Bioline USA, Inc.) and examined by quantitative real-time PCR (qPCR). qPCR was accomplished on a QuantStudio 3 qPCR machine (Applied Biosystems) using FastSYBR Green (Thermo Fisher) and 10 nM concentrations of primers designed using PrimerDesign (Thermo Fisher). The relative fold change was calculated with the 18S rRNA housekeeping gene using the ΔΔ*C_T_* method.

gDNA was extracted from mouse stool using the Zymo gDNA isolation kit (Zymo) according to the manufacturer’s instructors with the addition of two rounds of bead beating. To generate a standard curve for comparison, F. nucleatum was grown overnight in BHIS, and 1 ml was serial diluted and used to isolated gDNA. These same cultures were plated for CFU counts, generating matching gDNA and CFU values. gDNA from mouse stool and culture standards were examined using FAST SYBR green and primers (*Fusobacterium* forward, CAACCATTACTTTAACTCTACCATGTTCA; *Fusobacterium* reverse, GTTGACTTTACAGAAGGAGATTATGTAAAAATC) on a QuantStudio3 qPCR machine. The *Fusobacterium* load was calculated based on the cycle of threshold (*C_T_*) values of the standards and back-calculated to CFU by using a four-parameter logistics curve as previously described (112).

### Statistics.

Data are presented as means ± the standard deviations, with points representing individual mice. Comparisons between groups were made with the Student *t* test or one- or two-way analysis of variance (ANOVA), using the Holm-Sidak *post hoc* test. GraphPad was used to generate graphs and statistics (GraphPad Software, Inc., La Jolla, CA). A *P* value of <0.05 was considered significant, and “*n*” indicates the number of experiments performed.
